# Disparate selection of mutations in the dihydrofolate reductase gene (*dhfr*) of *Plasmodium ovale curtisi* and *P*. *o*. *wallikeri* in Africa

**DOI:** 10.1371/journal.pntd.0010977

**Published:** 2022-12-05

**Authors:** Jing Chen, Xiaoqin Ma, Jianxia Tang, Sui Xu, Yaping Gu, Feng Tang, Yuanyuan Cao, Weiming Wang, Huayun Zhou, Jiayao Zhang, Xinyu Yu, Guoding Zhu, Meng Zhu, Qingfeng Zhang, Richard Culleton, Yaobao Liu, Jun Cao

**Affiliations:** 1 National Health Commission Key Laboratory of Parasitic Disease Control and Prevention, Jiangsu Provincial Key Laboratory on Parasite and Vector Control Technology, Jiangsu Institute of Parasitic Diseases, Wuxi, China; 2 Center for Global Health, School of Public Health, Nanjing Medical University, Nanjing, China; 3 Laboratory of Molecular Parasitology, Key Laboratory of Spine and Spinal Cord Injury Repair and Regeneration of Ministry of Education, Tongji Hospital; Clinical Center for Brain and Spinal Cord Research, School of Medicine, Tongji University, Shanghai, China; 4 Division of Molecular Parasitology, Proteo-Science Centre, Ehime University, Matsuyama, Ehime, Japan; University of Liverpool, UNITED KINGDOM

## Abstract

*Plasmodium ovale curtisi* and *P*. *ovale wallikeri* are both endemic in sub-Saharan Africa, the Middle East and Southeast Asia. Molecular surveillance data for drug resistance in *P*. *ovale spp*. is limited at present. We analysed polymorphisms in the *podhfr*, *pocrt* and *pocytb* genes of *P*. *ovale spp*. in 147 samples collected from travelers returning to China from Africa. Two *podhfr* mutations, S58R and S113N/T were detected in *P*. *ovale curtisi* with high/moderate frequencies of 52.17% and 17.39%, respectively. Evidence of positive selection (d_N_/d_S_ = 2.41) was found for *podhfr* in *P*. *ovale curtisi* and decreased diversity (*H*_*e*_) of microsatellite markers flanking the mutant alleles suggests that selective sweeps have occurred for both. Mutations E34G (1.50%) and L43V (1.50%) in *pocrt* of *P*. *ovale curtisi*, and E34G (3.70%), I102M (1.80%) and V111F (1.80%) of *P*. *ovale wallikeri* were found at low frequencies. Mutations R66K (6.20%), R75K (11.63%) and R95K (3.88%) of *pocytb* were found in both *P*. *ovale curtisi* and *P*. *ovale wallikeri*. These results suggest that the *podhfr* gene of *P*. *ovale curtisi* may be subject to drug selection in Africa, warranting further attention. We observed significant differences in the prevalence and distribution of *podhfr* mutations between the two *P*. *ovale* species, suggestive of fundamental biological differences between them.

## Author summary

The importance of the hitherto neglected malaria parasites *Plasmodium ovale curtisi*, *Plasmodium ovale wallikeri* and *Plasmodium malariae* is now becoming apparent. It is clear that these species are prevalent in Africa at much higher rates than previously thought, and that they are responsible for a significant amount of malaria-related morbidity. There is very little currently known about the epidemiology of these parasites, despite their importance. Of particular importance is understanding whether these parasites undergo selection for drug resistance in similar ways as *Plasmodium falciparum* and *Plasmodium vivax*. The fact that little is known regarding the molecular epidemiology of these parasites is due to difficulties in identifying them in the field, the fact that they are often present at low parasite densities in the blood, are often masked by co-infection with *P*. *falciparum*, and that there is no *in vitro* culture system available for them. We were able to utilise a unique and priceless resource–*P*. *ovale spp*. samples, mostly in mono-infections, from non-immune Chinese travellers to Africa–to sequence orthologues of several genes known to be linked to drug resistance in other malaria parasite species. This resource enabled us to sample a number of parasites from a widespread geographical area; something that has never before been possible. Through sequencing of the orthologues of several genes known to be linked to drug resistance in other malaria parasite species, high frequencies of S58R and S113N/T mutations were observed in *podhfr* in *P*. *ovale curtisi* isolates from Africa. Furthermore, by surveying the genetic diversity around these loci using microsatellite markers, we were able to show that the *podhfr* gene of African *P*. *ovale curtisi* has undergone selection for particular mutations, probably due to drug pressure, and this warrants further attention.

## Introduction

Malaria remains a major global public health threat with an estimated 241 million cases and 627,000 deaths reported worldwide in 2020, with more than 90% of these cases occurring in Africa[1]. There are six species of malaria parasite that commonly cause malaria in humans: *Plasmodium falciparum*, *Plasmodium vivax*, *Plasmodium malariae*, *Plasmodium knowlesi*, *Plasmodium ovale curtisi and Plasmodium ovale wallikeri*. The latter two parasites were previously considered to be a single species, *P*. *ovale*, but are now known to be separate species. The prevalence of *P*. *ovale spp*. is probably underestimated due to low parasite densities and difficulties in detection by microscopy [2, 3].

Global malaria control and elimination has made great progress. There remain, however, many challenges among which antimalarial drug resistance is of major concern [4]. Malaria parasite resistance to antimalarial drugs manifests in delayed or incomplete clearance of parasites during their proliferation in the blood of the host. This reduction in susceptibility allows drug tolerant and resistant parasites to persist in the blood post-treatment, where they may produce gametocytes capable of infecting mosquitoes.

Multi-drug resistant strains of both *P*. *falciparum* and *P*. *vivax* have been described. Following the emergence of Chloroquine (CQ) resistant *P*. *falciparum*, sulfadoxine-pyrimethamine (SP) was used extensively as a safe and inexpensive alternative first-line treatment in many malaria-endemic areas [5]. However, use of this drug has been hampered by the development and spread of drug resistance [6], and it has been replaced by artemisinin combination therapies (ACTs) in most countries. Despite this, the WHO still recommends its use as an intermittent preventive treatment during pregnancy (IPTp) and in infants (IPTi) in sub-Saharan Africa [7].

SP belongs to the antifolate drug class which target the folate-synthesis pathway of *Plasmodium* parasites. Dihydrofolate reductase (DHFR) plays an important role in the synthesis of folic acid by malaria parasite [8]. Pyrimethamine binds to DHFR, competitively excluding its natural substrate, dihydrofolate [8]. Point mutations in the *P*. *falciparum* DHFR gene (*dhfr*) reduce the affinity between DHFR and pyrimethamine, reducing the latter’s ability to inhibit folic acid synthesis, leading to parasite resistance to the drug [8]. Three mutations N51I, C59R, and S108N in *pfdhfr* are now widespread in Africa [9].

Although SP is not and never has been recommended to treat patients with *P*. *ovale spp*. malaria, it is possible that the presence of the drug in populations in which both *P*. *falciparum* and *P*. *ovale spp*. are prevalent may select for resistance in the latter parasite by proxy [10].

At present, *in vitro* culture of *P*. *ovale spp*. is not possible, and *in vivo* drug efficacy testing is very difficult. The only viable approach to assaying whether *P*. *ovale spp*. parasites may have developed resistance against anti-malarial drugs is to detect the prevalence of mutations in orthologs of genes associated with drug resistance in other *Plasmodium* species. Currently, molecular investigations of drug resistance markers in *P*. *ovale spp*. are limited. We analyzed the prevalence of genetic polymorphisms in *podhfr*,*pocrt* and *pocytb*, the orthologues of which are known to be associated with drug resistance in *P*. *falciparum*, in *P*. *ovale spp*. samples collected from imported malaria cases returning from Africa to China.

## Methods

### Ethics statement

This study was reviewed and approved by the Institutional Ethics Committee of Jiangsu Institute of Parasitic Diseases (JIPD). The written informed consent was obtained from all participants.

### Sample and data collection

Whole blood samples were collected from *P*. *ovale spp*. positive migrant workers returning to Jiangsu province of China from Africa between 2012 and 2016. Jiangsu Province is located in east China with only imported malaria cases reported since 2012 [11]. Case epidemiological information was collected from the web-based China Routine Disease Surveillance Information System (CRDSIS) and Jiangsu Provincial Malaria Weekly [12]. *Plasmodium ovale spp*. genomic DNA was extracted from 200μl whole blood using the QIAamp DNA Mini Kit (Qiagen Valencia, CA, USA) according to the manufacturer’s protocol. *Plasmodium* infection was initially diagnosed by microscopy and confirmed by species-specific nested PCR [13] in Jiangsu Provincial Reference Laboratory of Malaria Diagnosis. Only *P*. *ovale spp*. single species infections were included in this study. *Plasmodium ovale spp*. samples were genotyped to discriminate between of *P*. *ovale curtisi* and *P*. *ovale wallikeri* using Real-time PCR as previously described [14].

### Genotyping of *podhfr*, *pocrt* and *pocytb*

The *podhfr* gene was amplified by nested PCR, the *pocrt* and *pocytb* genes were amplified by single round PCR (primer sequences and PCR conditions are provided in S1 Table). Amplified products were analyzed by 1% agarose gel electrophoresis and the PCR products were sent to Tianlin Biotechnology (Shanghai) company for Sanger sequencing. Polymorphisms were identified with reference to *P*. *ovale curtisi dhfr* (*pocdhfr*: LT594586.1), *P*. *ovale wallikeri dhfr* (*powdhfr*: LT594509.1), *P*. *ovale curtisi crt* (*poccrt*: PocGH01_01016900), *P*. *ovale wallikeri crt* (*powcrt*: LT594505.1) and *P*. *ovale cytb* (*pocytb*: LT594520.1). Sequences were aligned using BioEdit Version7.2 [15] and point mutations were identified using DNAstar Version7.1.0.

### *Podhfr*-flanking microsatellite analysis

Five microsatellite loci flanking the *podhfr* gene (upstream: -47kb, -33.5kb; downstream: 0.3kb, 33.1kb and 39.2kb) were used to identify potential selective sweeps associated with the spread of drug resistant alleles of *podhfr*. Microsatellite loci within a flanking region of 100kb around the *podhfr* gene of *P*. *ovale curtisi* (*pocdhfr*) were screened using Tandem Repeats Finder [16]. Fluorescently labeled primers and PCR conditions for microsatellite genotyping are provided in S2 Table. Products labelled with Hex fluorophore were separated and detected on an ABI 3730 capillary sequencer (Thermo Fisher Scientific, US) by Sangon Biotech (Shanghai, China). DNA fragment sizes were determined and visualized using GeneMapper software Version 3.2 (Applied Biosystems, Foster City, CA, USA). The dominant allele (highest intensity peak) at each locus, and any additional alleles with peak height ≥ 33% of the dominant allele peak heights of > 200 rfu (fluorescence units) were scored [17]. Genotyping success was defined as the presence of at least one allele at a given locus in a given sample.

### Population genetics analysis

The number of single nucleotide polymorphisms (SNPs), average pairwise nucleotide diversity (π), haplotype diversity (*Hd*) and number of haplotypes (H) were calculated using DnaSP Version5.10.01 [18]. The presence of selection was analyzed by comparing the ratio of non-synonymous to synonymous mutations. Tajima’s D, Fu and Li’s D*, and Fu and Li’s F* tests were analyzed to measure the degree of deviation from neutrality using DnaSP Version5.10.01 [18]. The rates of non-synonymous mutations (d_N_) and synonymous mutations sites (d_S_) were computed by Z-tests based on the Nei & Gojobori method with the Jukes and Cantor correction and 1000 bootstrap replications using MEGA Version6.06 [19]. The diversity of microsatellites flanking *podhfr* was estimated by the expected heterozygosity (*H*_*e*_) using GenAlEx Version6.5 [20], where *H*_*e*_ = [n/(n–1)](1–Σp_i_^2^) and n represents the number of isolates and P_i_ is the frequency of the ith allele. Microsatellite polymorphism parameters including allele number and expected heterozygosity (*H*_*e*_) were calculated for different geographical regions and mutant strains. Mann-Whitney U and Wilcoxon rank sum tests were used to determine the significance of differences in *H*_*e*_ (*P*<0.05). Pair-wise *F*_*st*_ was employed as a proxy of the genetic distance between pairs of populations and was calculated using Arlequin Version 3.5[21].

## Results

### Origin of *Plasmodium ovale* samples

A total of 147 laboratory-confirmed *P*. *ovale spp*. samples from 18 African countries were collected from 2012 to 2016 (Table 1). The majority of samples were from Central Africa (80/147, 54.4%), Southern Africa (35/147, 23.8%) and West Africa (30/147, 20.4%) with the top three countries being Equatorial Guinea (n = 49), Angola (n = 30) and Nigeria (n = 19). Of the 147 samples, 138, 121 and 129 samples were successfully genotyped for *podhfr* (*P*. *ovale curtisi* = 69, *P*. *ovale wallikeri* = 69), *pocrt* (*P*. *ovale curtisi* = 67, *P*. *ovale wallikeri* = 54) and *pocytb* (*P*. *ovale curtisi* = 55, *P*. *ovale wallikeri* = 74), respectively (Table 2).

**Table 1:** Geographic origins of *P*. *ovale spp*. samples.

Regions	Countries	*P*. *ovale curtisi*	*P*. *ovale wallikeri*	Total
**Southern Africa**	**Angola**	9	21	30
	**Mozambique**	1	2	3
	**South Africa**	1	0	1
	**Zambia**	1	0	1
**West Africa**	**Guinea**	1	3	4
	**Ghana**	1	1	2
	**Cote d’Ivoire**	0	1	1
	**Liberia**	2	0	2
	**Niger**	1	0	1
	**Nigeria**	11	8	19
	**Sierra Leone**	0	1	1
**Central Africa**	**Equatorial Guinea**	28	21	49
	**Republic of Congo**	6	7	13
	**Democratic Republic of the Congo**	5	1	6
	**Gabon**	1	3	4
	**Cameroon**	5	3	8
**East Africa**	**Uganda**	0	1	1
**North Africa**	**South Sudan**	0	1	1
**Total**		**73**	**74**	**147**

**Table 2:** Mutations of *podhfr*, *pocrt* and *pocytb* in *P*. *ovale curtisi* and *P*. *ovale wallikeri* isolates.

Genes	Mutations	*P*. *ovale curtisi*	*P*. *ovale wallikeri*	Total
***podhfr***	**K48R**	1.44%(1/69)	0%(0/69)	0.72%(1/138)
	**F57L**	0%(0/69)	5.80%(4/69)	2.90%(4/138)
	**S58R**	52.1%(36/69)	5.80%(4/69)	28.99%(40/138)
	**T62R**	0%(0/69)	2.9%(2/69)	1.45%(2/138)
	**P98H**	1.44%(1/69)	0%(0/69)	0.72%(1/138)
	**S113T**	2.9%(2/69)	1.44%(1/69)	2.17%(3/138)
	**S113N**	14.5%(10/69)	2.9%(2/69)	1.45%(2/138)
	**I169T**	0%(0/69)	1.44%(1/69)	0.72%(1/138)
	**E342D**	1.44%(1/69)	0%(0/69)	0.72%(1/138)
***pocrt***	**E34G**	1.50%(1/67)	3.70%(2/54)	2.48%(3/121)
	**L43V**	1.50%(1/67)	0%(0/54)	0.83%(1/121)
	**I102M**	0%(0/67)	1.80%(1/54)	0.83%(1/121)
	**V111F**	0%(0/67)	1.80%(1/54)	0.83%(1/121)
***pocytb***	**R66K**	7.27%(4/55)	5.41%(4/74)	6.20%(8/129)
	**Z67K**	1.81%(1/55)	0%(0/74)	0.78%(1/129)
	**R75K**	12.73%(7/55)	10.81%(8/74)	11.63%(15/129)
	**R95K**	3.64%(2/55)	4.05%(3/74)	3.88%(5/129)

### Polymorphisms in *podhfr*, *pocrt* and *pocytb*

**Fig 1:**
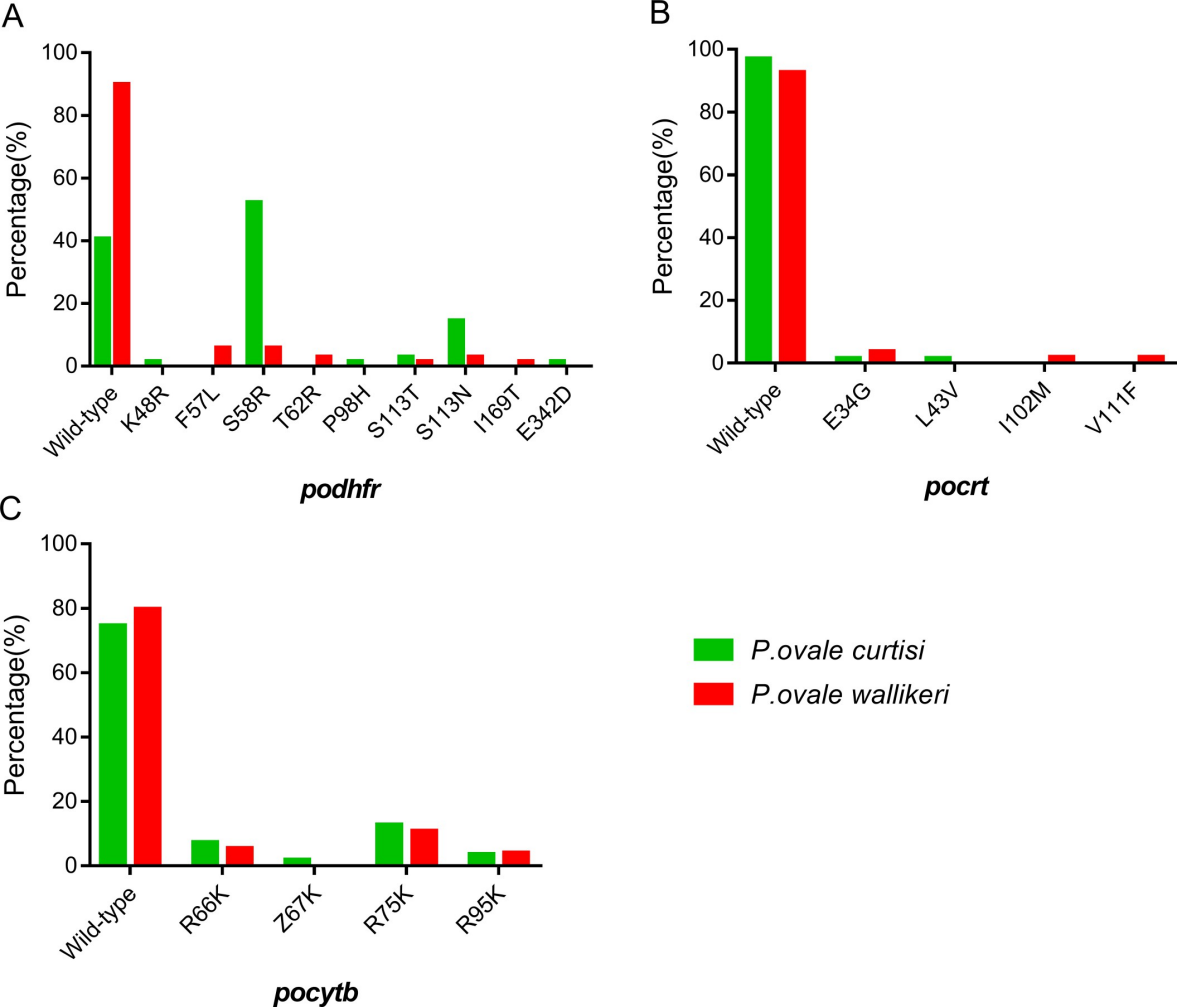
Mutation prevalence of *podhfr*, *pocrt* and *pocytb* in *P*. *ovale curtisi* and *P*. *ovale wallikeri* isolates. (A) *podhfr* (B) *pocrt* (C) *pocytb*.

Nine distinct mutations were found in *podhfr*, among which the most common were S58R (36/69, 52.1%) and S113N (10/69, 14.5%) with higher frequencies observed in *P*. *ovale curtisi* than *P*. *ovale wallikeri* (Table 2 and Fig 1A). Most of the S58R mutations (32/36, 88.9%) in *P*. *ovale curtisi* were from Central Africa, among which 20 were from Equatorial Guinea, five were from the Democratic Republic of the Congo, four were from the Republic of Congo and three were from Cameroon. The frequency of the S58R mutation in Central Africa (74.4%, 32/43) was significantly higher than West Africa (14.3%, 2/14) and Southern Africa (16.7%, 2/12). Most of the S113N mutations (8/10, 80%) in *P*. *ovale curtisi* were also from Central Africa with five samples from the Democratic Republic of the Congo and three samples from the Republic of Congo. The frequency of S113N mutation in Central Africa (18.6%, 8/43) was slightly higher than Southern Africa (16.7%, 2/12). All 10 samples of *P*. *ovale curtisi* with S113N mutations were S58R/S113N double mutants. Seven and five *podhfr* haplotypes were found with haplotype diversities (*Hd*) of 0.681 and 0.192 in *P*. *ovale curtisi* and *P*. *ovale wallikeri*, respectively (Table 3).

For *pocrt*, four mutations were found, all at low frequencies. E34G and L43V were observed in only one sample each, and only in *P*. *ovale curtisi*.E34G was observed in two samples of *P*. *ovale wallikeri*. I102M and V111F were observed in only one sample each and only in *P*. *ovale wallikeri* (Table 2 and Fig 1B). For *pocytb*, four mutations were found, with R75K present at the highest frequency of 12.73% in *P*. *ovale curtisi* and 10.81% in *P*. *ovale wallikeri* (Table 2 and Fig 1C). More details regarding the population genetics of *pocrt* and *pocytb* are given in Table 3.

**Table 3:** Population genetics parameters of the *podhfr*, *pocrt* and *pocytb* in *P*. *ovale* curtisi and *P*. *ovale* wallikeri isolates.

Genes	Subspecies	n	H	π	*Hd*	SNP	N	S	d_N_	d_S_	d_N_ /d_S_	Tajima’s D test	Fu and Li test
***podhfr***	***P*. *ovale curtisi***	69	7	0.00173	0.68	7	6	1	0.00198	0.00082	2.41	-0.585	-1.2555
	***P*. *ovale wallikeri***	69	5	0.00062	0.19	6	6	0	0.00078	0	-	-1.6405	-0.6645
***pocrt***	***P*. *ovale curtisi***	67	3	0.00028	0.06	2	2	0	0.00043	0	-	-1.43018	-2.66318
	***P*. *ovale wallikeri***	54	4	0.00059	0.14	4	3	0	0.0004	0	-	-1.57792	-1.75144
***pocytb***	***P*. *ovale curtisi***	55	8	0.00066	0.39	7	4	3	0.00029	0.00107	0.276	-2.8076	-6.7526
	***P*. *ovale wallikeri***	74	9	0.00059	0.34	5	3	2	0.00082	0	-	-2.7958	-7.7833

n, number of isolates; H, number of distinct haplotypes; π, nucleotide diversity; N, number of non-synonymous mutations; S, number of synonymous mutations.

### Differences in *dhfr* mutations between *P*. *ovale wallikeri* and *P*. *ovale curtisi*

**Fig 2:**
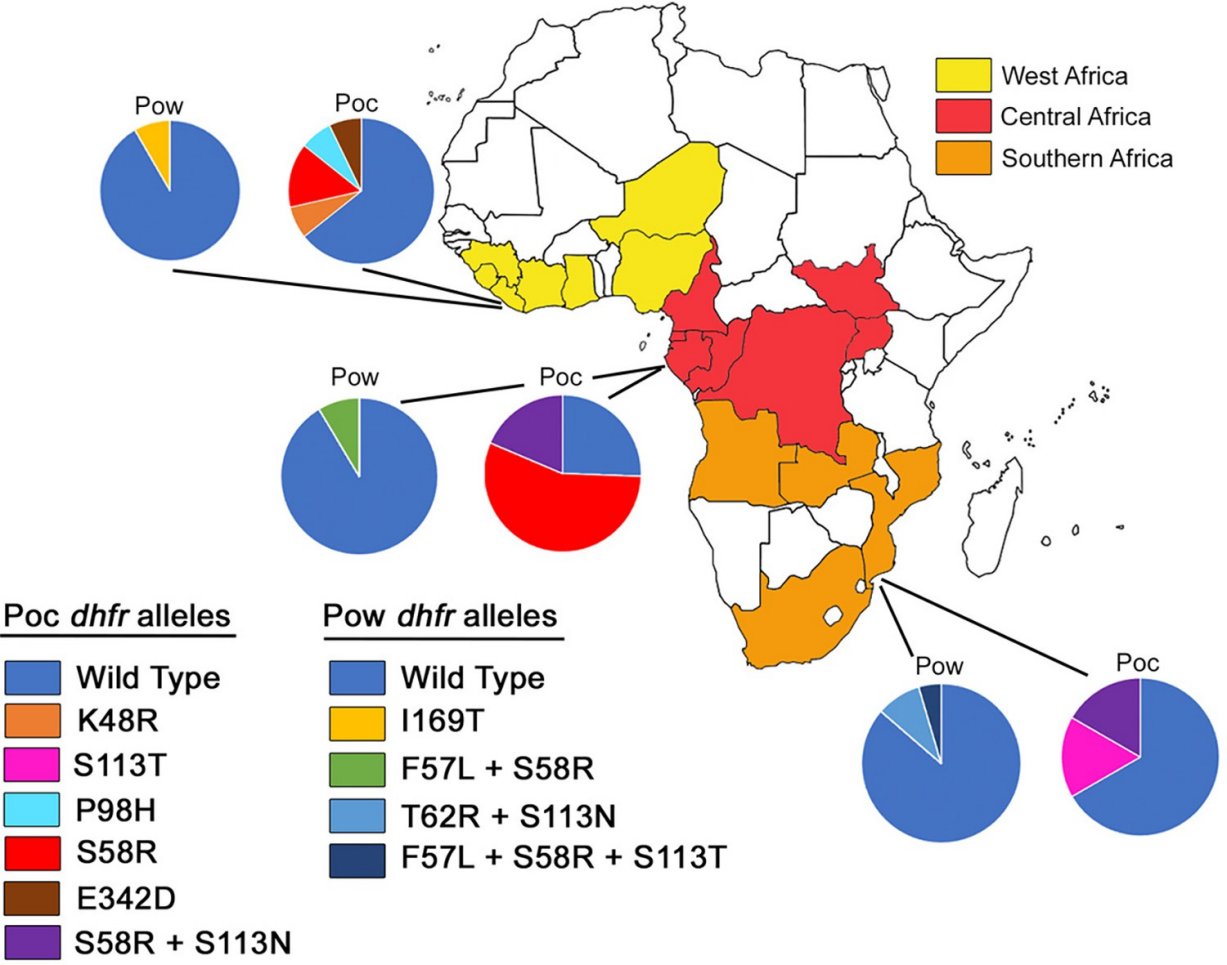
Geographical origin of *Plasmodium ovale curtisi* (Poc) and *P*. *ovale wallikeri* (Pow) isolates, and the proportion of mutant and wild type alleles of the dihydrofolate reductase gene (*dhfr*) from each region. Countries from which isolates originated are coloured according to region (West, Central or Southern Africa). The link to the base layer of the map is https://freesvg.org/laurent-afrique-politique.

Fig 2 shows the proportion of mutant alleles of *P*. *ovale curtisi* and *P*. *ovale wallikeri* in isolates from West, Central and Southern Africa. The individual mutations S58R and S113N were observed in both species. In *P*. *ovale curtisi*, S58R was observed both as a single mutation and in combination with S113N, whereas in *P*. *ovale wallikeri*, S58R only appeared in combination with F57L in a double mutant allele, and with F57L and S113T as part of a triple mutant allele. All other mutations were unique to one or other of the species. The *P*. *ovale curtisi* population from Central Africa was the only one in which mutant alleles were more prevalent than wild types, with 74% (32/43) of isolates carrying either S58R single mutations or S58R + S113N double mutations. The former allele was also observed (at lower prevalence) in both West and Southern Africa, making it the most geographically widespread mutant allele. Mutant alleles were present at a prevalence of 59% (41/69) in *P*. *ovale curtisi*, and 10% (7/69) of *P*. *ovale wallikeri* isolates.

### Selective sweeps involving *podhfr* mutations in *P*. *ovale curtisi*

**Fig 3:**
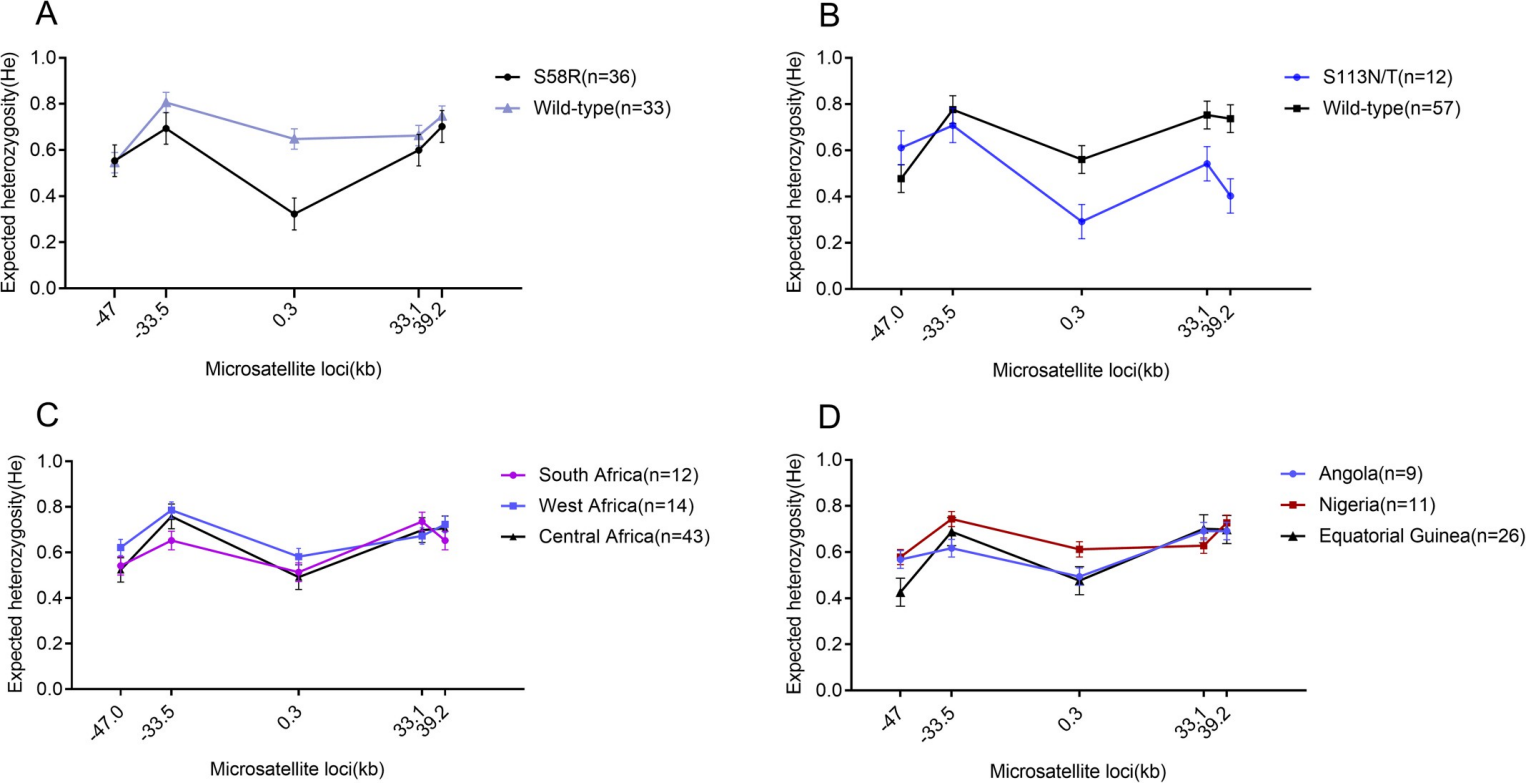
The expected heterozygosity *(H*_*e*_) of microsatellite loci flanking *podhfr* gene in *P*. *ovale curtisi*. (A) S58R mutant strains vs wild-type strains. (B) S113N/T mutant strains vs wild-type strains. (C) Comparison in different regions in Africa. (D) Comparison in different countries in Africa.

The d_N_/d_S_ ratio for *P*. *ovale curtisi podhfr* was 2.41, indicating the presence of positive selection pressure. *pocrt* and *pocytb* did not appear to be under the influence of positive selection, and there were no significant differences in neutral evolutionary statistics (Table 3). Based on analysis of five microsatellite loci, the genetic diversity of *podhfr* S58R mutant strains (*H*_*e*_ = 0.575±0.069) was significantly lower than that of wild-type strains (*H*_*e*_ = 0.682±0.045, *P* = 0.011) in *P*. *ovale curtisi* (Fig 3A and S4 Table). The genetic diversity of S113N/T mutant strains (*H*_*e*_ = 0.511±0.074,) was significantly lower than that of wild-type strains (*H*_*e*_ = 0.661±0.060, *P* = 0.001) in *P*. *ovale curtisi* (Fig 3B and S4 Table). This decreased genetic diversity in loci surrounding mutant alleles suggests a selective sweep has occurred involving *podhfr* gene mutations in *P*. *ovale curtisi*. The number of alleles for each microsatellite marker in *P*. *ovale spp*. isolates of each population is shown in S3 Table. Microsatellite polymorphism was relatively similar in different regions / countries in Africa (Fig 3C and 3D and S4 Table).

The genetic differentiation coefficients of *P*. *ovale spp*. strains among Southern Africa, West Africa and Central Africa differed. At the regional level, the genetic differentiation between West Africa and Central Africa (*F*_*st*_ = 0.038, *P* >0.05) was the highest (S5 Table). At the country level, the genetic differentiation between Nigeria and Equatorial Guinea (*F*_*st*_ = 0.041, *P* >0.05) was the highest (S6 Table). There was significant genetic differentiation between S58R mutant strains and the wild-type strains (*F*_*st*_ = 0.103, *P*<0.05) (S7 Table), and between the S113N/T mutant strains and the wild-type strains (*F*_*st*_ = 0. 087, *P*<0.05) (S8 Table).

## Discussion

We analyzed genetic polymorphisms in *P*. *ovale dhfr*, *crt* and *cytb* in 147 samples collected from imported cases of malaria in migrant workers returning to China from Africa. These imported cases were of non-immune migrant workers (and not short-term travelers), who had resided in African countries for a relatively long periods of time, which enabled us to sample a large number of parasites from a widespread geographical area. Furthermore, these workers spent extended time periods in Africa, and would have experienced correspondingly longer exposure times than short-term travelers.

Overall, a low level of genetic polymorphism in these drug resistance-related genes were observed. We observed the highest level of polymorphism in the *dhfr* gene (π = 0.00118), and this polymorphism was significantly higher in *P*. *ovale curtisi* (π = 0.00173) compared to *P*. *ovale wallikeri* (π = 0.00062). Non-synonymous mutations at the 58th and 113th amino acids positions of *dhfr* were observed in both *P*. *ovale spp*. sub-species. Furthermore, S58R and S113N were detected in *P*. *ovale curtisi* with high/moderate frequencies of 52.17% and 17.39%, respectively, but were rare in *P*. *ovale wallikerii* which may be related to biological differences between the species that lead to differing susceptibilities to drugs. These differences may inform different public health policies targeting these two *P*. *ovale spp*. species. The S58R mutation has also been reported in other *Plasmodium* species. For example, three mutations (F57L, S58R, S117N) in *P*. *vivax* were found with high frequency in cases from Thailand and were considered to be associated with high levels of SP resistance through patient follow-up [22]. Seven mutations in *dhfr* (K55E, S58R, S59A, F168S, N194S, D207G and T221A) were also identified in 123 samples of *P*. *malariae* from Asia and Africa [23].

It is now established that the parasite species previously known as ‘*Plasmodium ovale’* is in fact two separate species, which, despite their clear separate species status are still referred to with the sub-species names *P*. *ovale wallikeri* and *P*. *ovale curtisi*. They are indistinguishable by microscopy, but have been shown to constitute sympatric and non-recombining species. Their ranges overlap, and they are sometimes found in co-infections of the same individual. To date, there is little evidence of major differences between the species phenotypically[24], but genotypic studies indicate distinct genetic lineages with little evidence of introgression[25]. In this context, our findings of distinct mutation profiles in the *dhfr* gene of both species, and of significantly greater prevalence of mutations in *P*. *ovale curtisi* compared to *P*. *ovale wallikeri*, perhaps constitute the strongest evidence to date of an obvious biological difference between the species. Striking differences were observed between the species at the *dhfr* locus, with no allelic types in common between the species, and with a much larger proportion of *P*. *ovale curtisi* isolates carrying mutations compared to those of *P*. *ovale wallikeri*. These differences were particularly obvious when considering isolates from Central Africa, where the majority of *P*. *ovale curtisi* isolates carried mutated *dhfr* alleles, whereas in *P*. *ovale wallikeri* isolated from the same region, parasites carrying wild-type isolates were dominant.

As the two species have previously been shown to be sympatric in this region, we assume that both are likely to have been exposed to antifolate drugs to the same degree. Given this, several possible scenarios for the observed disparity between the species at the *dhfr* locus present themselves. Firstly, *P*. *ovale wallikeri* may develop resistance to antifolates at loci other than *dhfr*. Some drug resistance phenotypes in malaria parasites involve mutations in multiple genes, and some species develop resistance to the same drug through mutations at different loci (for example, resistance to CQ is mediated through mutations in the chloroquine transporter gene, *crt*, and the multidrug resistance gene *mdr1* in *P*. *falciparum*, but not in *P*. *vivax* or *P. chabaudi[26]*. However, a low prevalence of mutations was observed in the *dhfr* gene of *P*. *ovale wallikeri*, including those, such as S58R and S113N, the orthologues of which are known to confer antifolate resistance in other species, suggesting some conservation of resistance mechanisms in this species.

A second possible explanation for the relatively low prevalence of mutations in *dhfr* in *P*. *ovale wallikeri* compared to *P*. *ovale curtisi*, is that antifolate use in the host population results in less selective pressure for the acquisition of resistance in the former species compared to the latter. This may be due to inherent susceptibility differences between the species, for instance with *P*. *ovale wallikeri* being naturally more tolerant to pyrimethamine than *P*. *ovale curtisi*. This situation is not without precedent, as *Plasmodium yoelii*, the malaria parasite of African thicket rats, is known to be far more naturally resistant to chloroquine than its rodent malaria parasite cousin *P*. *chabaudi*[27] (both these species, like the *P*. *ovale spp*., naturally infect the same mammalian hosts).

Further explanations may include reduced exposure of *P*. *ovale wallikeri* to antifolates compared to *P*. *ovale curtisii*, perhaps due to range differences or to niche differences within the blood. Furthermore, given that antifolate treatment of *P*. *falciparum* is probably the main driver of resistance in *P*. *ovale spp*., it is possible that differences in the *P*. *ovale* species propensity to co-infect with *P*. *falciparum* may influence the acquisition of resistance mutations. Additionally, it is possible that *P*. *ovale wallikeri* may suffer from a greater fitness cost of *dhfr* mutations than *P*. *ovale curtisi*. Further studies are required to shed light on this phenomenon.

While the S58R mutation was most commonly observed in single mutation *pocdhfr* alleles, the S113N mutation was always found in combination with S58R, suggesting that the acquisition of pyrimethamine resistance in *P*. *ovale curtisi* may occur in a step-wise manner, with the S58R mutation necessary before S113N is selected. This mirrors the situation in *P*. *falciparum*, in which the *pfdhfr* S108N mutation is thought to be an essential first step prior to the acquisition of further mutations such as *pfdhfr* C59R. Of note, the order of selection is reversed in *P*. *ovale curtisi* compared to *P*. *falciparum*, with the *pocdhfr* S113N mutation (perhaps orthologous to *pfdhfr* S108N) following after the *podhfr* S58R mutation (orthologous to *pfdhfr* C59R). In *P*. *ovale curtisi*, the picture is somewhat different, with the S58R and F57L mutations only ever appearing together, and the S113N and S113T alleles appearing exclusively in combination with T62R and F57L + S58R, respectively. It should be stressed, however, that there is no experimental evidence linking the mutations described in *podhfr* to pyrimethamine resistance.

We found regional differences in the distribution of putative pyrimethamine resistance associated mutations in *P*. *ovale curtisi podhfr*. Both S58R single mutants and S58R + S113N mutations were more prevalent in Central Africa compared to west and southern Africa. Given the lack of polymorphism found in microsatellite markers linked to these alleles suggests a single origin and a subsequent selective sweep, it seems likely that these mutant alleles originated in Central Africa from where they are spreading to the rest of the continent. Further surveys of *P*. *ovale spp*. populations in Africa are required to test this hypothesis.

It should be noted that antifolates are no longer recommended as treatment for malaria in Africa, so it is possible that rather than observing an ongoing selection of resistance-associated mutations, we may actually be witnessing the reverse; the re-emergence and spread of wild-type parasites in the absence of antifolate drug pressure. However, it is impossible to assess the impact of ongoing antifolate use in the populations sampled here, either for non-malaria related reasons such as prophylaxis among HIV infected people, or as non-officially prescribed treatments for malaria. Clarifying this situation will require further sampling of the *P*. *ovale spp*. populations in the future, combined, perhaps, with retrospective analysis of archived samples.

The d_N_/d_S_ ratio of *P*. *ovale curtisi podhfr* in was 2.41, indicative of the action of directional selection on this gene. Previously, microsatellites markers have been used to measure the degree of genetic polymorphism at loci around genes linked to drug resistance, in order to identify potential ‘selective sweeps’, in which a particular allele of a gene becomes dominant in a population following its emergence in a single, or limited number of parasite(s). For example, a study involving the analysis of four microsatellite loci flanking the *dhfr* gene from 50 *P*. *falciparum* strains in Ghana revealed a selective sweep of the mutant allele probably driven by the use of SP [28]. In another study, twenty-six microsatellite markers flanking the *dhfr* gene were used to study *P*. *falciparum* strains collected in Southeastern Africa [29]. It was found that genetic diversity within 70 KB of the allele associated with the highest degree of resistance was significantly reduced; evidence of a selective sweep following the widespread use of SP.

In order to further explore whether the *podhfr* gene of *P*. *ovale curtisi* has been subjected to drug selection pressure, we analyzed the diversity of five microsatellite loci flanking the gene. Samples of *P*. *ovale curtisi* were divided into different populations based on their geographical origin and mutation sites, and their diversity analysed. We found that allele diversity did not differ significantly between populations, but there was a greater degree of diversity in microsatellite markers flanking wild-type alleles than those flanking mutant alleles.

There were no statistical differences in expected heterozygosity at each microsatellite locus between the three geographic regions. The expected heterozygosity of microsatellite loci flanking the S58R mutant allele was significantly lower than those flanking the wild-type allele. Similarly, the expected heterozygosity of markers flanking the S113N/T mutant was lower than those of the wild-type allele. This reduced heterozygosity around the mutant alleles is evidence selective sweeps, probably driven by exposure to antifolate drugs. Both the S58R and S113N/T mutations were most commonly found in Central and West Africa, which may be related to the drug policy in these regions (CQ was replaced by SP for intermittent preventive treatment in sub-Saharan African countries).

Point mutations at codons 72, 74, 75, and 76 in the *pfcrt* gene confer resistance to CQ in *P*. *falciparum* [30], and the mutations C101F, H97Y, F145I, M343L, G353V are associated with Piperaquine (PQ) and Amodiaquine (AMQ) resistance [31, 32]. At present, there are no reports on polymorphisms of the *pocrt* gene in *P*. *ovale spp*. We found four non-synonymous mutations, E34G, L43V, I102M and V111F in *pocrt*, all at low frequencies. Orthologs of these mutations have not been described in *P*. *falciparum*. Mutations in the *pfcytb* gene confer drug resistance to atovaquone-proguanil in *P*. *falciparum* [33]. In a previous study only one non-synonymous *cytb* mutation (M248I) was found in 19 *P*. *ovale spp*. samples from Africa [34]. We found three non-synonymous mutations, R66K, R75K and R95K, which were present at relatively high frequencies. The orthologue of the *P*. *falciparum* atravaquinone resistance associated mutation at position 268 was not found. Selection pressure analysis showed that the *pocytb* gene was under purifying selection (d_N_/d_S_ = 0.276) in *P*. *ovale curtisi*.

Although a relatively small number of samples were analysed in this study and sample sources are unevenly distributed throughout Africa, we found evidence of positive selection on *podhfr*, and of the signature of selective sweeps around the mutant alleles. Despite the fact that the mutations observed have orthologues in *P*. *falciparum* that are known to be associated with resistance against anti-folate drugs, there is currently no evidence to link the *P*. *ovale spp*. mutations to drug resistance. Further in vitro or in vivo drug assays are required to verify the involvement of these mutations in *P*. *ovale spp*. response to drug treatment.

## Conclusion

High frequencies of S58R and S113 N/T mutations were observed in *podhfr* in *P*. *ovale curtisi* isolates from Africa, and evidence of selective sweeps around these mutant loci were recorded. The *podhfr* gene in *P*. *ovale curtisi* may, therefore, have undergone selection pressure due to exposure to antifolate drugs. Further *P*. *ovale spp*. resistance surveillance in Africa is recommended.

## Supporting information

S1 Table Primers and PCR conditions for amplification of *podhfr*, *pocrt* and *pocytb*. (DOCX)

S2 Table Primers and PCR conditions for Microsatellite markers. (DOCX)

S3 Table The number of alleles for each microsatellite locus in *P*. *ovale curtisi* isolates. (DOCX)

S4 Table The expected heterozygosity (*H*_*e*_) of microsatellite loci flanking *podhfr* gene in *P*. *ovale curtisi*. (DOCX)

S5 Table Genetic differentiation (*F*_*st*_) of *P*. *ovale spp*. between different regions of Africa. (DOCX)

S6 Table Genetic differentiation (*F*_*st*_) of *P*. *ovale spp*. between different countries of Africa. (DOCX)

S7 Table Genetic differentiation (*F*_*st*_) between S58R mutant and wild-type isolates. (DOCX)

S8 Table Genetic differentiation (*F*_*st*_) between S113N/T mutant and wild-type isolates. (DOCX)
