# Supplementary material for: Disparate selection of mutations in the dihydrofolate reductase gene (dhfr) of Plasmodium ovale curtisi and P. o. wallikeri in Africa
Source: PLoS Negl Trop Dis. 2022 Dec 5;16(12):e0010977. doi: 10.1371/journal.pntd.0010977 (PMC9754596; doi:10.1371/journal.pntd.0010977)
Supplement: S1 Table — (DOCX) [file pntd.0010977.s001.docx]

**S1 Table. Primers and PCR conditions for amplification of *po*dhfr, *pocrt* and *pocytb.***

| **Genes** | **Primers** | **PCR Conditions** |
| --- | --- | --- |
| ***po*dhfr** | Podhfr-F1: 5'-CGCGATATGCGCGTGCTGCAAA-3'  Podhfr-R1: 5'-CTGGGTGGACATAATTCTCTCTAT -3'  Podhfr-F2: 5'-GAGATTGGAAGAAGAGAATCGT-3'  Podhfr-R2: 5'-TTCCGGGTTTAGTTTAAGCGTG-3' | Primary PCR: 94℃ for 2 min, 40 cycles (98℃ for 10 sec, 58℃ for 2 min, 68℃ for 2 min), 68℃ for 5 min.  Secondary PCR: 94℃ for 2 min, 35 cycles (98℃ for 10 sec, 54℃ for 30 sec, 68℃ for 1.5 min), 68℃ for 5 min. |
| ***pocrt*** | Pocrt-F: 5'-GGCCAAATTGAAGAAAGAACATCTA-3'  Poctr-R: 5'-GACGGTTTCGTGTAACGACTG-3' | 94°C for 5min,44 cycles (94°C for 60s, 51°C for 30s, 69°C for 50s), 60°C for 5min. |
| ***pocytb*** | Pocytb-F: 5'- AATGTTTGCTTGGGAGCT -3'  Pocytb-R: 5'- CATTTATTAAATTACCCATGTCC -3' | 94°C for 5min, 44 cycles (94°C for 60s, 51°C for 30s, 69°C for 50s), 60°C for 5min. |
