# Supplementary material for: Disparate selection of mutations in the dihydrofolate reductase gene (dhfr) of Plasmodium ovale curtisi and P. o. wallikeri in Africa
Source: PLoS Negl Trop Dis. 2022 Dec 5;16(12):e0010977. doi: 10.1371/journal.pntd.0010977 (PMC9754596; doi:10.1371/journal.pntd.0010977)
Supplement: S2 Table — (DOCX) [file pntd.0010977.s002.docx]

**S2 Table. Primers and PCR conditions for Microsatellite markers**

| **Markers** | **Primers** | **Sequence(5’-3’)** | **PCR Conditions** |
| --- | --- | --- | --- |
| **-47kb** | STR564-1F | ATGTCATCTGATAAATGGGAGG | 95°C for 3min,  10 cycles (95°C for 30s, 60°C for 30s, 72°C for 30s),  72°C for 5min. |
|  | STR564-1R | AATCACAAAGGTCAAATGCG |  |
| **-33.5kb** | STR564-3F | GAAGTAGAGAAGCGGAGTGGTAT |  |
|  | STR564-3R | GTTCTATTTCCCTTTTTCTGTCTC |  |
| **0.3kb** | STR564-6F | GAACGGTTCGTCTGAATGAG |  |
|  | STR564-6R | AAGGTGCATTTAAGCGGAT |  |
| **33.1kb** | STR564-8F | AACGTCTCTTATCAAGTTCATGG |  |
|  | STR564-8R | TTCTCCCTTACATTCAAAATACTTC |  |
| **39.2kb** | STR564-9F | TTCGAAGATTGCCAATTATGT |  |
|  | STR564-9R | CAAACGAAAAAGTTCAGATCAGT |  |
