# Supplementary material for: Disparate selection of mutations in the dihydrofolate reductase gene (dhfr) of Plasmodium ovale curtisi and P. o. wallikeri in Africa
Source: PLoS Negl Trop Dis. 2022 Dec 5;16(12):e0010977. doi: 10.1371/journal.pntd.0010977 (PMC9754596; doi:10.1371/journal.pntd.0010977)
Supplement: S3 Table — (DOCX) [file pntd.0010977.s003.docx]

**S3 Table. The number of alleles for each microsatellite locus in *P. ovale* *curtisi* isolates**

| **Populations** | **Microsatellite loci** | | | | | **Average** | **Total** |
| --- | --- | --- | --- | --- | --- | --- | --- |
|  | **MS1** | **MS3** | **MS6** | **MS8** | **MS9** |  |  |
| **S58R mutant(n=36)** | 3 | 7 | 3 | 4 | 4 | 4.2 | 21 |
| **Wild-type(n=33)** | 4 | 11 | 5 | 7 | 6 | 6.6 | 33 |
| **S113B/T mutant(n=12)** | 3 | 4 | 3 | 3 | 3 | 3.2 | 16 |
| **Wild-type(n=57)** | 4 | 12 | 5 | 7 | 5 | 6.6 | 33 |
| **Southern Africa(n=12)** | 3 | 4 | 4 | 5 | 4 | 4 | 20 |
| **Angola(n=9)** | 3 | 4 | 3 | 4 | 4 | 3.6 | 18 |
| **West Africa(n=14)** | 4 | 6 | 4 | 5 | 4 | 4.6 | 23 |
| **Nigeria(n=11)** | 3 | 5 | 4 | 4 | 4 | 4 | 20 |
| **Central Africa(n=43)** | 3 | 9 | 4 | 5 | 5 | 5.2 | 26 |
| **Equatorial Guinea(n=26)** | 2 | 5 | 3 | 5 | 4 | 3.8 | 19 |
| **Total number of alleles** | **5** | **13** | **5** | **7** | **6** | **7.2** | **19** |
