# Supplementary material for: Disparate selection of mutations in the dihydrofolate reductase gene (dhfr) of Plasmodium ovale curtisi and P. o. wallikeri in Africa
Source: PLoS Negl Trop Dis. 2022 Dec 5;16(12):e0010977. doi: 10.1371/journal.pntd.0010977 (PMC9754596; doi:10.1371/journal.pntd.0010977)
Supplement: S4 Table — (DOCX) [file pntd.0010977.s004.docx]

**S4 Table. The expected heterozygosity (*H_e_*) of microsatellite loci flanking *podhfr* gene in *P. ovale curtisi***

| **Types** | **Populations** | **MS1** | **MS3** | **MS6** | **MS8** | **MS9** | ***H_e_* (Mean±SE)** | ***P*-value** |
| --- | --- | --- | --- | --- | --- | --- | --- | --- |
| **Geographical origins** | Southern Africa | 0.542 | 0.653 | 0.514 | 0.736 | 0.653 | 0.619±0.041 |  |
|  | West Africa | 0.622 | 0.786 | 0.582 | 0.673 | 0.724 | 0.678±0.036 | 0.092 |
|  | Central Africa | 0.524 | 0.759 | 0.492 | 0.699 | 0.706 | 0.636±0.054 |  |
|  | Angola | 0.568 | 0.617 | 0.494 | 0.691 | 0.691 | 0.612±0.038 |  |
|  | Nigeria | 0.579 | 0.744 | 0.612 | 0.628 | 0.727 | 0.658±0.033 | 0.131 |
|  | Equatorial Guinea | 0.426 | 0.689 | 0.476 | 0.701 | 0.698 | 0.598±0.061 |  |
| **Amino acid sites** | S58R mutant | 0.554 | 0.694 | 0.323 | 0.600 | 0.702 | 0.575±0.069 | 0.011 |
|  | Wild-type | 0.545 | 0.806 | 0.648 | 0.663 | 0.747 | 0.682±0.045 |  |
|  | S113B/T mutant | 0.611 | 0.708 | 0.292 | 0.542 | 0.403 | 0.511±0.074 | 0.001 |
|  | Wild-type | 0.478 | 0.776 | 0.560 | 0.753 | 0.737 | 0.661±0.060 |  |
