# Supplementary material for: Disparate selection of mutations in the dihydrofolate reductase gene (dhfr) of Plasmodium ovale curtisi and P. o. wallikeri in Africa
Source: PLoS Negl Trop Dis. 2022 Dec 5;16(12):e0010977. doi: 10.1371/journal.pntd.0010977 (PMC9754596; doi:10.1371/journal.pntd.0010977)
Supplement: S5 Table — (DOCX) [file pntd.0010977.s005.docx]

**S5 Table. Genetic differentiation (*F_st_*) of *P. ovale* between different regions of Africa**

| **Regions** | **Southern Africa** | **West Africa** | **Central Africa** |
| --- | --- | --- | --- |
| **Southern Africa** | - |  |  |
| **West Africa** | 0.004(*P*=0.32) | - |  |
| **Central Africa** | -0.012(*P*=0.7) | 0.038(*P*=0.06) | - |
