# Supplementary material for: Disparate selection of mutations in the dihydrofolate reductase gene (dhfr) of Plasmodium ovale curtisi and P. o. wallikeri in Africa
Source: PLoS Negl Trop Dis. 2022 Dec 5;16(12):e0010977. doi: 10.1371/journal.pntd.0010977 (PMC9754596; doi:10.1371/journal.pntd.0010977)
Supplement: S6 Table — (DOCX) [file pntd.0010977.s006.docx]

**S6 Table. Genetic differentiation (*F_st_*) of *P. ovale* between different countries of Africa**

| **Countries** | **Angola** | **Nigeria** | **Equatorial Guinea** |
| --- | --- | --- | --- |
| **Angola** | - |  |  |
| **Nigeria** | -0.023 (*P*=0.6) | - |  |
| **Equatorial Guinea** | 0.025 (*P*=0.26) | 0.041(*P*=0.06) | - |
