# Supplementary material for: Disparate selection of mutations in the dihydrofolate reductase gene (dhfr) of Plasmodium ovale curtisi and P. o. wallikeri in Africa
Source: PLoS Negl Trop Dis. 2022 Dec 5;16(12):e0010977. doi: 10.1371/journal.pntd.0010977 (PMC9754596; doi:10.1371/journal.pntd.0010977)
Supplement: S8 Table — (DOCX) [file pntd.0010977.s008.docx]

**S8 Table. Genetic differentiation (*F_st_*) between S113N/T mutant and wild-type isolates**

| **Type** | **S113N/T mutant** | **Wild-type** |
| --- | --- | --- |
| **S113N/T mutant** | - |  |
| **Wild-type** | 0.087 (P<0.05) | - |
